# Soft Ionic Electroactive Polymer Actuators with Tunable Non-Linear Angular Deformation

**DOI:** 10.3390/ma10060664

**Published:** 2017-06-21

**Authors:** Wangyujue Hong, Abdallah Almomani, Yuanfen Chen, Reihaneh Jamshidi, Reza Montazami

**Affiliations:** 1 Department of Mechanical Engineering, Iowa State University, Ames, IA 50011, USA; hwyj@iastate.edu (W.H.); yuanfenc@iastate.edu (Y.C.); reihaneh@iastate.edu (R.J.); 2 Department of Aerospace Engineering, Iowa State University, Ames, IA 50011, USA; almomani@iastate.edu; 3 Department of Energy, Ames Laboratory, Ames, IA 50011, USA

**Keywords:** ionic electroactive polymers, electromechanical actuators, soft materials, angular deformation

## Abstract

The most rational approach to fabricate soft robotics is the implementation of soft actuators. Conventional soft electromechanical actuators exhibit linear or circular deformation, based on their design. This study presents the use of conjugated polymers, Poly(3,4-ethylenedioxythiophene)-poly(styrenesulfonate) (PEDOT:PSS) to locally vary ion permeability of the ionic electroactive polymer actuators and manipulate ion motion through means of structural design to realize intrinsic angular deformation. Such angular deformations are closer to biomimetic systems and have potential applications in bio-robotics. Electrochemical studies reveal that the mechanism of actuation is mainly associated with the charging of electric double layer (EDL) capacitors by ion accumulation and the PEDOT:PSS layer’s expansion by ion interchange and penetration. Dependence of actuator deformation on structural design is studied experimentally and conclusions are verified by analytical and finite element method modeling. The results suggest that the ion-material interactions are considerably dominated by the design of the drop-cast PEDOT:PSS on Nafion.

## 1. Introduction

The field of robotics is currently dominated by “hard robots” consisting of hard materials, mainly metallic or composite structures, paired with either (or both) ceramic actuators or electric motors as drive trains. Although hard robots sometimes have biomimetic design and limb-like structures similar to those in animals (e.g., “Big Dog” constructed by Boston Robotics [1]), hard robots often use wheels and rotary motors for motion, which distance them from biomimetic design and deters their integration with biomimetic systems. Soft actuators, on the other hand, have enabled soft robotics that can move and be manipulated, exhibiting biomimetic physical and mechanical attributes similar to those of Mollusca [2,3,4,5,6]. The ultimate advantages of soft actuators are that (1) they can easily conform to curvilinear structures, like biological muscles; and (2) since actuation is an intrinsic property of the actuator, micro-scale systems are practical to design and fabricate.

Electroactive polymer actuators, and in particular ionic electroactive polymer (IEAP) actuators, have attracted enormous interest and attention from the soft-robotic community and have been subject to extensive studies over the past several years [7,8,9,10,11,12,13]. Depending on the design, IEAP actuators can exhibit either linear or circular deformation. Linear IEAP actuators have a minuscule electromechanical response, which is not adequate for locomotion; circular deformation, however, is substantial. IEAP actuators consist of an ionomeric membrane at the core, covered with conductive network composite (CNC) layers and metal electrodes on each side to enhance ionic mobility and electric conductivity. IEAP actuators are doped with electrolytes, typically ionic liquids, to provide the ion-rich environment required for actuation. IEAP actuators’ performance and attributes depend on many factors, including the thickness and chemical structure of the ionomeric membrane [14]; the thickness, density, porosity, and electric conductivity of CNC layers [15,16,17,18,19]; the thickness and electric conductivity of metal electrodes [20]; and the type, mobility, and prevalence of mobile ions [14,17,21,22,23]. IEAP actuators exhibit two physical deformations: cationic and anionic. Under an applied electric field cations and anions compete to reach the electrodes of opposite charge. There is often (depending on chemical composition of the electrolyte [24], CNC, and ionomeric membrane) a time lag between the accumulation of different ions at the electrodes. Therefore, in our case the actuator initially bends toward anode, which is due to accumulation of cations and called cationic deformation, then follows a bending toward cathode, which is due to accumulation of anions and called anionic deformation. This behavior is previously explored and discussed in detail [15,16,17,18,19].

Circular-bending soft actuators can be used to mimic a Venus flytrap [25], flap wings [26,27], create artificial muscles [12,28], and propel fish robots [29,30]. But, circular actuation can also be considered a disadvantage of IEAP actuators concerning soft bio-robotic applications, as it is distinctly different from most biological systems. Although vertebrates and invertebrates have many muscles with circular or sinusoidal motion (e.g., tongue, abdominal muscles, etc.), they are not used for locomotion. Locomotor muscles are usually integrated with skeleton or exoskeleton structures to form limbs. Some robotics applications like microgrippers [8,31] and miniaturized five fingered robots [7] would have worked more efficiently with angular (limb-like) rather than circular motion. To achieve angular motion, some researchers controlled individual segments of the IEAP actuators, moving them in different directions [32,33]. A snake-like swimming robot is one example of this technique [34].

In this work, IEAP actuators with angular deformation, mimicking the limb-like motion in biological systems, are presented and studied. The limb-like motion is achieved intrinsically and without utilization of skeleton-like structures. Patterns of a conjugated polymer were deposited on the ionomeric membrane to introduce regions with selective ion permeability to manipulate deformation. Poly(3,4-ethylenedioxythiophene)-poly(styrenesulfonate) (PEDOT:PSS) was used in fabrication of patterns because of its high conductivity and facile processing [35,36,37,38,39,40,41,42,43]. Dependence of deformation on the patterns of conjugated polymers is studied as well as morphological asymmetry in patterns and their influence on the cationic and anionic deformations. Electromechanical and electrochemical studies are accompanied and verified by analytical and finite element method (FEM) modeling. This study is expected to provide a cornerstone for utilization of advanced manufacturing techniques such as 3D printing in fabrication of soft actuators [44,45].

## 2. Experimental

### 2.1. Materials

Commercially available Nafion membrane, 25 µm thick, (Ion Power, Inc., New castle, DE, USA) was used as the ionomeric membrane; 1-ethyl-3-methylimidazolium trifluoromethanesulfonate (EMI-Tf, molecular formula: C_7_H_11_F_3_N_2_O_3_S) ionic liquid (Sigma Aldrich, St. Louis, MO, USA) was used as received; poly(3,4-ethylenedioxythiophene)-poly(styrenesulfonate) (PEDOT:PSS) (3.0–4.0% in H_2_O, high-conductivity grade) (Sigma Aldrich, St. Louis, MO, USA) was diluted by mixing with de-ionized (DI) water at 1:1 ratio and was used for fabrication of conductive polymer patterns. Gold leaf, 50 nm thick, (24K, transfer, LA Gold Leaf, Azusa, CA, USA) was used as the outer electrodes.

### 2.2. Sample Fabrication

**Figure 1:**
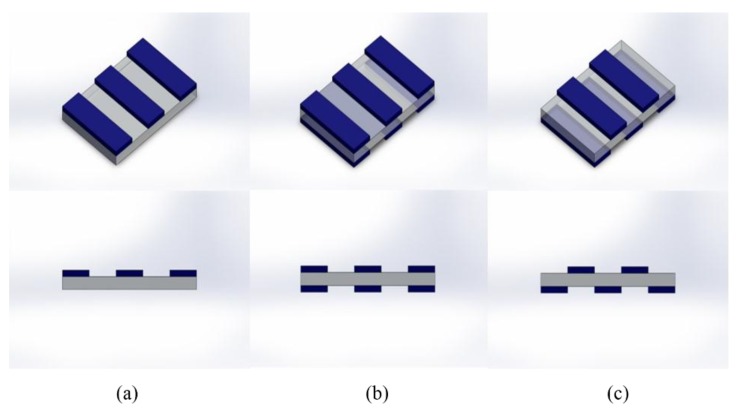
Angled (top row) and side (bottom row) views of patterned samples; (**a**) 1S, (**b**) 2SS, and (**c**) 2SA. Gold leaf electrodes are not shown in the sketch to give a better view of the patterns. Not to scale.

Nafion, in its acidic form, was first cut and soaked in EMI-Tf at 80 °C for 30 min to intake ~25 wt % of ionic liquid. Ionic liquid content was calculated as the weight percentage (wt %) of the dry weight of the membrane using Equation (1):

(1) $$W_{e}\;\left(\%\right)=\frac{W_{f}-W_{d}}{W_{f}}\times 100$$

where *W_e_* (%) is the weight percent of the electrolyte; and *W_d_* and *W_f_* are the weights of dry and doped Nafion, respectively [23]. The doped Nafion membrane was then placed between two sheets of filter paper overnight to flatten. Diluted PEDOT:PSS aqueous solution was drop-cast on the Nafion membrane over a vinyl mask at the concentration rate of 0.56 μL/mm^2^. Schematic representations of each pattern are provided in Figure 1. The coated and uncoated strips are equal in width and 3 mm each. The drop-cast patterns were dried slowly on a hot plate at 40 °C for 48 h to allow for complete solvent (DI water) evaporation, and prevent anisotropic shrinkage of samples. Employing this method, flat and smooth samples were obtained and no anisotropic shrinkage was observed. The coated samples were then dried under vacuum at 60 mmHg at room temperature for 24 h for further dehydration. Gold leaf electrodes were then hot-pressed at 95 °C under 1000 lb_f_ for 40 s on both sides of the membrane to form an IEAP actuator.

### 2.3. Sample Nomenclature

Sample nomenclature is shown in Table 1.

### 2.4. Electrochemical Characterizations

Impedance spectroscopy, current flow, and cyclic voltammetry studies were conducted on a VersaSTAT-4 potentiostat (Princeton Applied Research, Oak Ridge, TN, USA) in two-electrode mode. The impedance spectroscopy studies were carried out at frequencies between 1.0 × 10^5^ Hz and 1.0 × 10^−1^ Hz, and a potential difference (*ΔV*) of 10 mV. Current flow was studied as a function of ±4 V step function, each over a 600 s interval. Cyclic voltammetry was carried out in ±4 V potential window at a scan rate of 50 mV/s.

### 2.5. Electromechanical Characterizations

Actuators of 1 × 15 mm^2^ dimension were cut perpendicularly to the longitudinal direction of the PEDOT:PSS strips. Electromechanical responses of actuators as a function of a 4 V step function were monitored and recorded using a charge-coupled device (CCD) video camera, mounted to an in-house-constructed microprobe station, at 30 frames per second.

### 2.6. Morphological and Mechanical Characterizations

**Figure 2:**
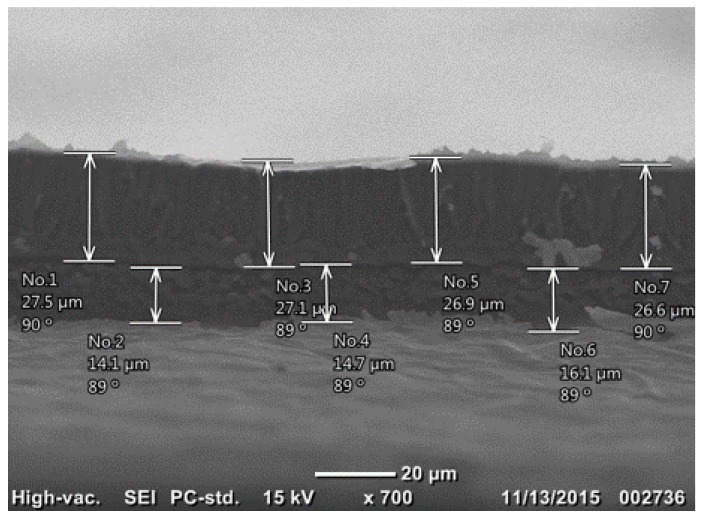
SEM image of the cross-section of specimen Nafion/1s-PEDOT:PSS/Au, indicating well interlayer adhesion between layers.

Surface analysis was conducted using scanning electron microscopy (SEM) (Jeol. JCM-6000 NeoScope, Peabody, MA, USA) to characterize morphology of the specimens, including film thickness and layer adhesion. The SEM image of the cross-section of Nafion/1s-PEDOT:PSS/Au sample is shown in Figure 2, indicating well interlayer adhesion between Nafion membrane and PEDOT:PSS layer after hot-press, with no observable separation in between.

The elastic modulus of each constituent layer of IEAP actuator was measured/calculated using a dynamic mechanical analyzer (DMA-1, Mettler Toledo, Columbus, OH, USA), loaded with tension clamps and operated in static modes. More information is included in Section 1, Supporting Information.

### 2.7. Finite Element Modeling

ABAQUS finite element software (ABAQUS/CAE 2016, Dassault Systèmes Simulia Corp., Johnston, RI, USA) was used to model the electromechanical responses of IEAP actuators and study the effect of various patterns on the actuation performance, and verify the experimental results. Details of the simulation procedures are presented in Section 2 of the Supporting Information.

## 3. Results

### 3.1. Cyclic Voltammetry

**Figure 3:**
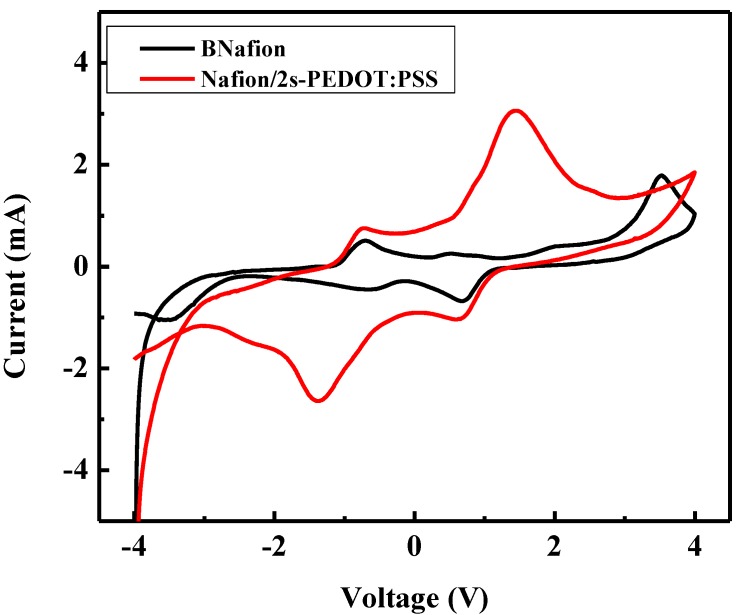
Cyclic voltammograms of different specimens measured at 50 mV/s.

Cyclic voltammograms of bare and coated specimens obtained in a potential range of ±4 V at a sweep rate of 50 mV/s are shown in Figure 3. Both voltammograms of specimens BNafion and Nafion/2s-PEDOT:PSS show reversible curves, revealing a reversible redox reaction at the electrode. When compared with BNafion, two additional significant current peaks are observed at ±1.5 V for Nafion/2s-PEDOT:PSS which are characteristic to PEDOT:PSS [38]. Additionally, another pair of current peaks are observed at ±0.8 V for both bare and coated Nafion, which correspond to ion drift across the Nafion membrane [46].

### 3.2. Equivalent Circuit Modeling

**Figure 4:**
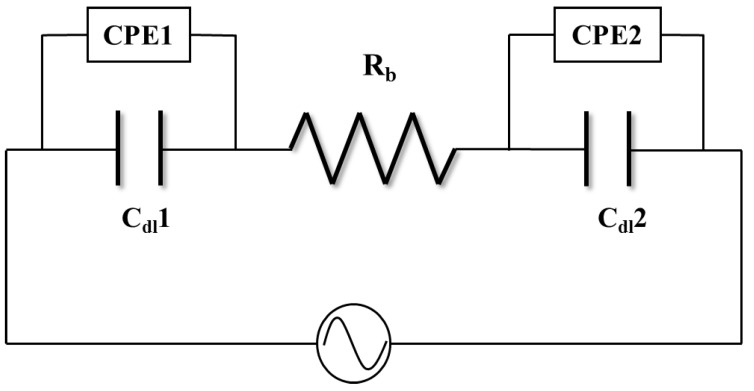
Equivalent circuit with a constant phase element.

Electric double layer (EDL) formation in IEAP actuators is a known phenomenon well-studied by others and us as well [17,18,19,22,38,47]. To investigate how the presence of PEDOT:PSS layers affect the formation of the EDL at the electrode, electrical impedance was studied as a function of frequency. Electrochemical studies were conducted at 10 mV and at a varying frequency to allow characterization over a broader frequency range. The electrochemical behavior of the system can be analyzed by fitting the electrical impedance with an equivalent electric circuit; as authors have previously shown for similar actuators with metallic colloid coatings [22,23,48]. The EDL capacitors in series with the resistance of bulk Nafion layer *R_b_* can be used to model the electrochemical behavior of such systems. The continuous contribution of a diffuse layer makes the pseudo-EDL capacitor very different from an ideal capacitor. Therefore, a constant phase element (CPE) was introduced in parallel with the EDL capacitor, as shown in Figure 4. The impedance of the introduced CPE, $W_{CPE}$, is defined as:

(2) $$W_{CPE}=\frac{1}{Y_{0}}{\left(j\omega \right)}^{-n}$$

where *ω* is angular frequency, and the property of a CPE is defined by two values, $Y_{0}$ and n. n is a unitless exponent taking values between 0 and 1. As n=0, CPE is identical to a resistor with $Y_{0}=1/R$, and when n=1, CPE is identical to a capacitor with $Y_{0}=C$.

**Figure 5:**
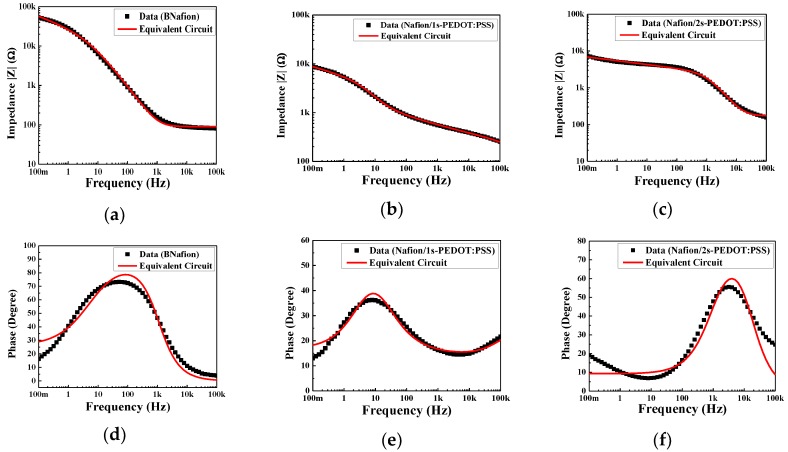
Impedance magnitudes of (**a**) BNafion, (**b**) Nafion/1s-PEDOT:PSS, and (**c**) Nafion/2s-PEDOT:PSS; and phase of (**d**) BNafion, (**e**) Nafion/1s-PEDOT:PSS, and (**f**) Nafion/2s-PEDOT:PSS fitted by equivalent circuit with constant phase element shown in Figure 4.

Considering the symmetric structure of BNafion and Nafion/2s-PEDOT:PSS, the impedance of two EDL capacitors and CPEs were set equally, that is, $C_{dl}1=C_{dl}2$, and $W_{CPE1}=W_{CPE2}$. Figure 5a–f present the experimental data and the fittings (solid curves) of the electric impedance magnitudes and phase angles of BNafion, Nafion/1s-PEDOT:PSS, and Nafion/2s-PEDOT:PSS. The model and the impedance spectrum in the entire frequency range are in good agreement. Table 2 summarizes the fitting parameters for the three actuators. The bulk membrane resistance, *R_b_*, is found to increase from 89.9 Ω (BNafion) to 171.7 Ω (Nafion/2s-PEDOT:PSS), primarily due to the indirect contact between the ionomer and the external electrode [9]. The PEDOT:PSS layers on both sides of Nafion/2s-PEDOT:PSS cause a significant drop in the capacitance of the EDL capacitor *C_dl_* compared to BNafion (from 3.24 to 0.12 µF).

In samples with the PEDOT:PSS layer casted only on one side of the Nafion membrane, an asymmetric charging behavior is induced by its morphological asymmetry. As presented in Table 2, the capacitance *C_dl_* improved on one side, while it dropped on the other side. The largest (12.78 µF) and smallest (3.52 × 10^−3^ µF) capacitances of *C_dl_* both occurred in the same specimen, but at different electrodes, indicating a highly imbalanced storage of ions at the external electrodes. The higher capability of the ions storage could happen in either the uncoated or PEDOT:PSS coated side, which will be discussed in the next sections with other experimental results.

### 3.3. Charging and Discharging

**Figure 6:**
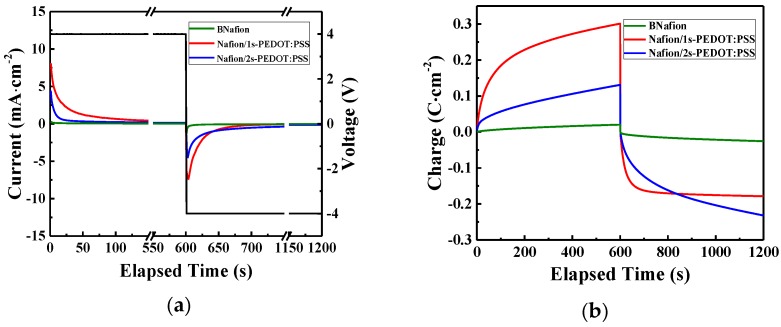
(**a**) Charging/discharging currents and (**b**) charge density versus time for different specimens under one cycle of a 4 V square wave.

To further investigate how the morphological asymmetry affects the charging/discharging behavior under a step voltage, current flow corresponding to a 4 V potential difference between the external electrodes was measured and recorded as a function of time (Figure 6a). Figure 6b presents the corresponding charge density stored in the specimen as a function of time. Each step function was set to 600 s; a much larger time range for the strain generated in these actuators that have already reached saturation [47]. The side with the casted PEDOT:PSS layer in Nafion/1s-PEDOT:PSS is connected to the working electrode, which experiences a higher potential in the charging process and a lower potential in the following discharging process. According to the cyclic voltammetry results, an electrochemical reaction occurs in the PEDOT:PSS layer when it is under a ±4 V voltage. The larger charging/discharging current in Nafion/2s-PEDOT:PSS (compared with BNafion) is due to the inserted/ejected electrons and the corresponding ion interchange. The charge density (area under the curve) difference between charging and discharging of symmetric samples (BNafion and Nafion/2s-PEDOT:PSS) is attributed to the random distribution of ions when charging is initiated compare to when discharging is initiated where, hypothetically, all ions are at the opposite electrode. While discharging, ions are traveling a longer path-length to reach the matching electrode, and this results in a larger current density. Additionally, a significantly larger magnitude of displaced charge (area under the curve) was observed in Nafion/1s-PEDOT:PSS. The highest charge density was observed when the PEDOT:PSS layer was connected to the higher potential in the charging process (Figure 6b, red curve, 0–600 s). This was followed by a less significant charge density during the discharging process (same plot, 600–1200 s). This phenomenon can be partially explained by the promoted ions’ drift, due to the better contact between the ionomer and the external electrode on the uncoated side. When at the higher potential, electrons will be ejected from PEDOT and positive charge carriers will be introduced to make the material electrically conducting, while at the lower potential, PEDOT will be reduced and partially loses its conductivity [49]. This also can explain the slightly decreased charge density of Nafion/1s-PEDOT:PSS during the discharging process. Overall Nafion/1s-PEDOT:PSS reveals the highest charge storage capacity.

### 3.4. Electromechanical Response

**Figure 7:**
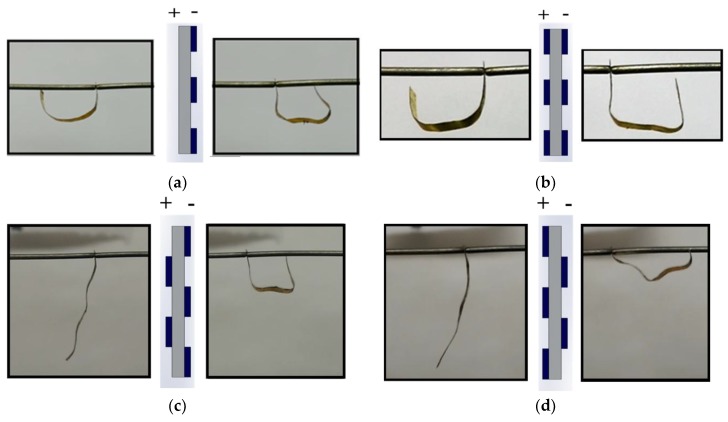
Schematic representation and experimental actuation performance for (**a**) 1S, (**b**) 2SS, (**c**) 2SA2, and (**d**) 2SA3. Left picture is the cationic response and right picture is the anionic response.

The electromechanical response of IEAP actuators with different PEDOT:PSS patterns was studied. Actuator 1S was first tested under a 4 V step function with the cathode connected to the PEDOT:PSS coated side. The cationic response was homogenous and circular toward the uncoated side. This behavior is similar to that of the actuators consisting of uniform CNC layers as reported previously [17,18,19,22,47]. However, as time progressed, this uniform actuation was canceled by the dominating anionic strain, which consists of a sharp, angular bending. Thus, the actuator exhibited a limb-like deformation. The schematic representation of the pattern and the images of the experimental results are shown in Figure 7a.

To further explore how different patterns affect the actuation performance, a 4 V step function was applied to the other two actuators, 2SS and 2SA; experimental results are presented in Figure 7b–d. The electromechanical response of the asymmetric 2SA actuator was studied under different polarities, 2SA2, and 2SA3. The 2SS actuator exhibited a rectangular, limb-like, deformation in both cationic and anionic deformations (Figure 7b), while 2SA actuators exhibited a more complex behavior, indicating a dependency on the electrode polarity (Figure 7c,d). In anionic motion, 2SA2 deformed into rectangle-like shape when actuator 2SA3 deformed into a triangle-like shape. Meanwhile, both cases have noticeable anionic deformation (strain) but almost negligible cationic deformation.

## 4. Discussion and Simulation

### 4.1. Discussion

Our experimental results suggest that the PEDOT:PSS layers as the CNC considerably affect the actuation behavior.

First, impedance data and the corresponding equivalent circuit modeling indicate that in Nafion/1s-PEDOT:PSS, ions are more likely to accumulate and act at the interface of one electrode, while depleted on the interface of the other electrode. However, when the PEDOT:PSS layer is casted on both sides of Nafion, a completely different phenomenon occurs and fewer ions move to charge the EDL capacitors. Consequently, fewer ions will accumulate at the outer electrodes; which in turn hinders mechanical deformation of the actuator.

Secondly, Nafion/1s-PEDOT:PSS exhibits the highest charge density under a 4 V square function. Since the main cause of the actuation is the accumulation and depletion of charged ions at the interfaces of the electrodes, the existence of PEDOT:PSS layer casted on only one side of Nafion would, most likely, enhance the actuation.

Additionally, electromechanical responses suggest that the actuation performance varies significantly with the existence of the PEDOT:PSS layer. Experimental results for actuators 1S, 2SA2, and 2SA3 all reveal an enhancement in the strain generation when the PEDOT:PSS layer only exists on the convex side, and an inhibition when it only exists on the concave side. Before the application of an electric potential, EMI-Tf ions are only distributed in the Nafion membrane, and presumably none in the PEDOT:PSS layer. Cyclic voltammetry results reveal that an electrochemical redox occurs in the PEDOT:PSS layer at ±1.5 V. Therefore, this enhancement-on-convex and inhibition-on-concave phenomenon may be caused by: (i) the expansion of the PEDOT:PSS layer due to the ion interchange and penetration to maintain charge neutrality; and (ii) ion accumulation and/or depletion at the electrodes. Experimental results for actuator 2SS, however, exhibit a completely reversed trend, that no matter whether in the convex or concave side, the PEDOT:PSS layer always hinders the actuation. That is, the existence of the PEDOT:PSS layer does not contribute considerably to expansion. Although, charging results reveal a relatively larger charging density in Nafion/2s-PEDOT:PSS than in BNafion, due to the electrochemical redox of PEDOT layers, this phenomenon could be explained by fewer ions moving to charge the EDL capacitors at the interfaces of electrodes.

Considering the structures of the actuators investigated in this study, segments of the actuators can be categorized under three possible structures: (1) uncoated membrane (BNafion); (2) single-side coated membrane (Nafion/1s-PEDOT:PSS); and (3) double-side coated membrane (Nafion/2s-PEDOT:PSS). Following scrutinizing the electromechanical response of actuators on the segment scale, it is concluded that: (1) for asymmetric segments, volume expansion occurs in the PEDOT:PSS layer due to ion interchange and ion accumulation/depletion at the interfaces of external electrodes; (2) the PEDOT:PSS layer does not contract; and (3) for symmetric segments, volume expansion occurs, but fewer ions move toward the electrodes than that of the uncoated segment. Therefore, deformation is enhanced on the uncoated segments and hindered on the coated segments.

### 4.2. Finite Element Simulation

**Figure 8:**
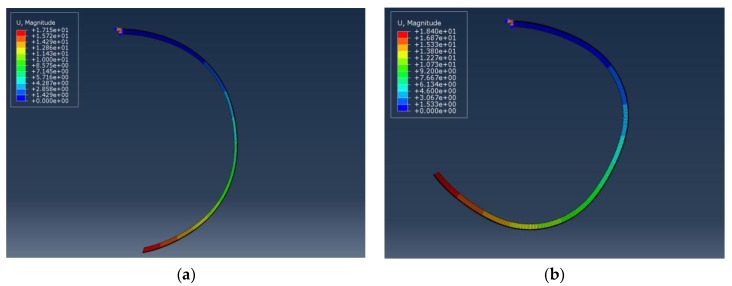
Displacement distribution of actuator 1S during cationic response under different hypotheses. When the total number of the movable ions in the actuator is fixed, the volume ratios of cations in the PEDOT:PSS layer (attached to the cathode) and the Nafion sub-layer (Supporting Information) are (**a**) 2:1 and (**b**) 1:2, respectively.

The conclusions drawn from experimental observations (Section 4.1) were examined and verified by FEM static analyses. The electromechanical response of IEAP actuators with different CNC patterns was modeled on ABAQUS/CAE using FEM (Section 2, Supporting Information). Figure 8 shows two different hypotheses for actuator 1S’s displacement distribution during cationic response; those are, cations are mainly accumulated in the PEDOT:PSS layer Figure 8a or Nafion Figure 8b. It confirms that in asymmetric segments, deformation mainly occurs on the PEDOT:PSS layer during the cationic response. Otherwise, instead of a homogeneous and circular deformation, a rectangular deformation occurs, which does not match the experimental results. It confirms that the largest capability of ions storage in Nafion/1s-PEDOT:PSS should locate at the coated side.

**Figure 9:**
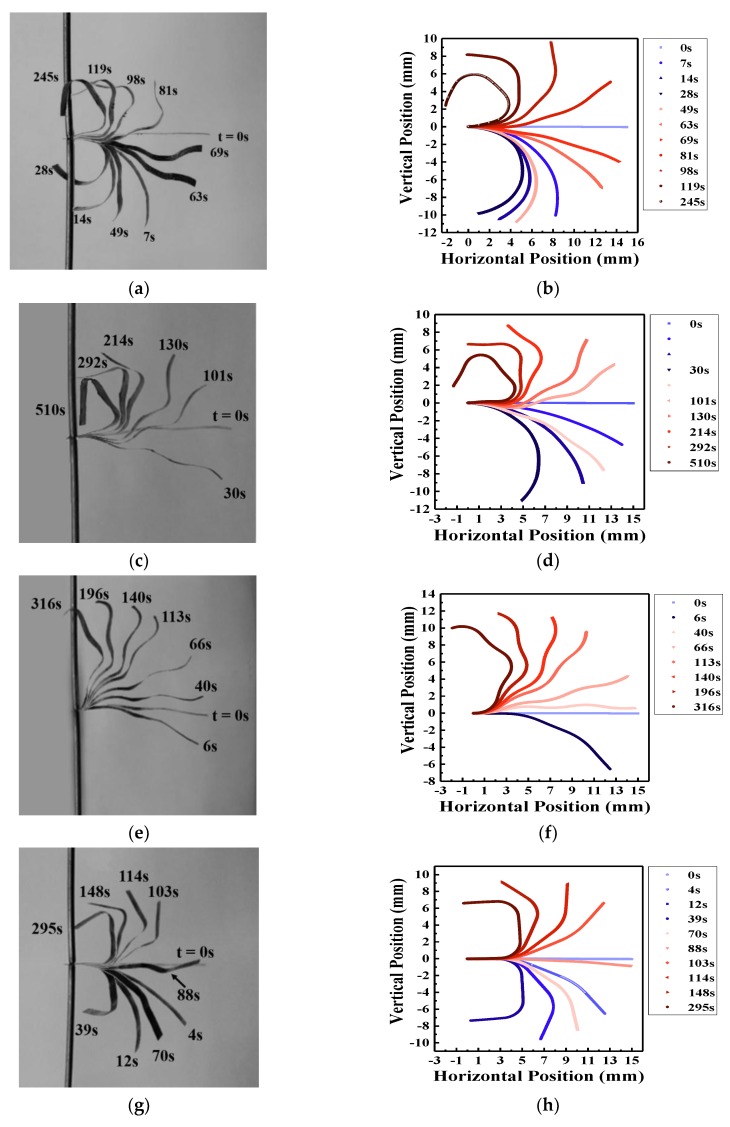
Comparison of experimental bending displacement in response to a 4 V step input (left column) and the corresponding results produced by the static theoretical model via ABAQUS (right column). Figure (**a**) and (**b**) represent actuator 1S, (**c**) and (**d**) represent actuator 2SA2, (**e**) and (**f**) represent actuator 2SA3, and (**g**) and (**h**) represent actuator 2SS. The top electrode is the cathode and the bottom electrode is the anode.

Figure 9 presents overlay images of experimental (Figure 9a,c,e,g) and the corresponding simulated (Figure 9b,d,f,h) results. Experimental results are collected under a 4 V step function and figures are extracted from video recordings. Simulations are the corresponding increments from static steps where blue and red gradation represents cationic and anionic strains, respectively. Experimental and simulated data are in good agreement, verifying the conclusive remarks made in Section 4.1.

In addition, as suggested by Hou et al., a simple aggregation model happened when EMI-Tf ionic liquid was absorbed into an ionic polymer membrane (Nafion), indicating an excess of negatively charged triple ions, (${\mathrm{Tf}}^{-}$-${\mathrm{EMI}}^{+}$-${\mathrm{Tf}}^{-}$) [24]. Without loss of generality, let EMI-Tf in Nafion membrane is in the format of (EMI^+^) and (${\mathrm{Tf}}^{-}$-${\mathrm{EMI}}^{+}$-${\mathrm{Tf}}^{-}$). In an IEAP actuator, 15 mm × 1 mm (*l* × *w*) and EMI-Tf uptake ~24 wt %, the increased weight is around 1.91 × 10^−4^ g. With the molecular weight of 260.23 g/mol in EMI-Tf, the molecular from EMI-Tf is 7.35 × 10^−7^ mol = 4.42 × 10^17^. Therefore, the total mobile cations (EMI^+^) and anion/anionic cluster (${\mathrm{Tf}}^{-}$-${\mathrm{EMI}}^{+}$-${\mathrm{Tf}}^{-}$) should be half of the total molecules inside, which equals to 2.21 × 10^17^. Meanwhile, when *ΔT* × *α* is small, the change in volume by thermal expansion *ΔV* can be simplified to $3\alpha \cdot \Delta T\cdot V_{0}$, by excluding the higher orders, where $V_{0}$ is the volume before any expansion/contraction. Simulations shown Figure 9 confirm a change in volume *ΔV* = 1.83 × 10^−11^ m^3^ in cationic response. Given the molecular volume of cations (EMI^+^) as 182 Å [47], an order of magnitude estimation is that 1.0 × 10^17^ (EMI^+^) cations are expected to contribute to the cationic response, which is almost half of the mobile cations inside Nafion membrane. In other words, based on the experiments and simulations indicated in Figure 9, approximately half of the ions from EMI-Tf contribute to actuation.

Simulations shown in Figure 9 set the volume ratio of cations and anions/anionic clusters based on the results reported by Hou et al. They characterized the diffusion ratio $D_{cation}/D_{anion}$ of EMI-Tf ionic liquid inside Nafion membranes as a function of water content *χ_water_* [24]. They discovered that when 15–30 wt % EMI-Tf is absorbed in Nafion at very low water contents, the diffusion ratio falls in the range of 1.5–2.5. The diffusion coefficient *D* is inversely proportional to the size of diffusing particles as described by the Stokes-Einstein relation:

(3) $$D=kT/\left(c\eta r_{H}\right)$$

where *k* is the Boltzmann constant, *T* is absolute temperature, *c* is a constant factor depending on the shape and relative size of the diffusion particle to its surrounding fluid, *η* is fluid viscosity, and *r_H_* is the hydrodynamic radius of the diffusing particle [24,50]. Since the cations and anions/anionic clusters exist in the same thermodynamic phase, $D_{cation}/D_{anion}\;$equals the reciprocal of their hydrodynamic radii ratio, which is proportional to the cubic root of the ions’ volume distributed in the Nafion membrane. That is $D_{cation}/D_{anion}=r_{Hanion}/r_{Hcation}\sim {\left(V_{anion}/V_{cation}\right)}^{\frac{1}{3}}$. The volume ratio of cations and anions/anionic clusters set in the simulation falls in the range of 1.5^3^–1.7^3^, which is consistent with the results reported by Hou et al.

## 5. Conclusions

Intrinsic angular deformation of IEAP actuators was achieved by incorporating conjugated polymer, PEDOT:PSS, patterns in the structure of soft actuators. Electrochemical and electromechanical studies were performed and it was observed that instead of the homogeneous circular deformation exhibited by conventional IEAP actuators, ones with polymer patterns bend at specific locations on the actuator which resulted in apparently angular deformation with sharp angles of 90° and beyond. Electromechanical responses indicate that actuation performances are significantly affected by different polymer patterns. Meanwhile, according to an FEM static model, approximately half of the ions from EMI-Tf contribute to the actuation. With different patterns of PEDOT:PSS, deformation patterns can be manipulated and actuators whose behaviors are complex but intrinsically controllable can be fabricated.

## Figures and Tables

**Table 1:** Sample abbreviations and corresponding definitions.

**Samples for Electromechanical Characterizations**	
Name	Definition
1S	Pattern deposited on one side, Figure 1a
2SS	Symmetric patterns on both sides, Figure 1b
2SA	Asymmetric patterns on both sides, Figure 1c
2SA2	2SA sample with 2 strip-patterned side attached to anode
2SA3	2SA sample with 3 strip-patterned side attached to anode
**Samples for Electrochemical Characterizations**	
Name	Definition
BNafion	Bare Nafion doped with ionic liquid
Nafion/1s-PEDOT:PSS	PEDOT:PSS drop-cast on one side (full coverage, no pattern)
Nafion/2s-PEDOT:PSS	PEDOT:PSS drop-cast on both sides (full coverage, no pattern)
**Sample for Morphological Characterizations**	
Name	Definition
Nafion/1s-PEDOT:PSS/Au	PEDOT:PSS drop-cast on one side (full coverage, no pattern), with gold leaf electrodes hot-pressed on both sides

**Table 2:** Fitting parameters for different specimens.

Circuit Element		BNafion	Nafion/1s-PEDOT:PSS	Nafion/2s-PEDOT:PSS
$R_{b}\left(Ω\right)$		89.9	118.9	171.7
CPE1	$Y_{0}\;\left({Ω}^{-1}\cdot cm^{-2}\cdot s^{n}\right)$	3.90 × 10^−5^	1.95 × 10^−4^	3.12 × 10^−4^
	n	0.29	0.18	0.11
CPE2	$Y_{0}\;\left({Ω}^{-1}\cdot cm^{-2}\cdot s^{n}\right)$	3.90 × 10^−5^	3.50 × 10^−4^	3.12 × 10^−4^
	n	0.29	0.21	0.11
$C_{dl}1\;\left(µF\right)$		3.24	12.78	0.12
$C_{dl}2\;\left(µF\right)$		3.24	3.52 × 10^−3^	0.12

## Supplementary Materials

The following are available online at http://www.mdpi.com/1996-1944/10/6/664/s1, Figure S1: SEM images of specimen Nafion/1s-PEDOT:PSS/Au, from middle point to edge ((a)–(c)) of PEDOT:PSS layer, Figure S2: Schematic of a bilayer laminate for the characterization of the elastic modulus of individual layer, Table S1: The thickness of each layer in IEAP actuator and its physical properties.
