# Supplementary material for: Soft Ionic Electroactive Polymer Actuators with Tunable Non-Linear Angular Deformation
Source: Materials (Basel). 2017 Jun 21;10(6):664. doi: 10.3390/ma10060664 (PMC5554045; doi:10.3390/ma10060664)
Supplement: Supplementary file 1 [file materials-10-00664-s001.pdf]

Supporting Information

Soft Ionic Electroactive Actuators with Tunable Non-Linear Angular Deformation

Wangyujue Hong[1,†], Abdallah Almomani[2,†], Yuanfen Chen[1], Reihaneh Jamshidi[1], Reza Montazami[1,3,*]

[1] Department of Mechanical Engineering, Iowa State University, Ames IA 50011, USA
[2] Department of Aerospace Engineering, Iowa State University, Ames IA 50011, USA
[3] Ames Laboratory, Department of Energy, Ames, IA 50011, USA
[*] Corresponding Author: reza@iastate.edu; Tel: +1-515-294-8733
[†] These authors contributed equally to this work

Keywords: ionic electroactive polymers, electromechanical actuators, soft materials, angular deformation

1. Morphological and Mechanical Characterizations.

Figures S1a - S1c show a wedge-shaped thickness profile of the casted PEDOT:PSS layer. Similar to a trapezoid, the highest concentration is in the center, and gradually tapers off along the edge, due to the imperfect fabrication process of the simple drop-cast technique.

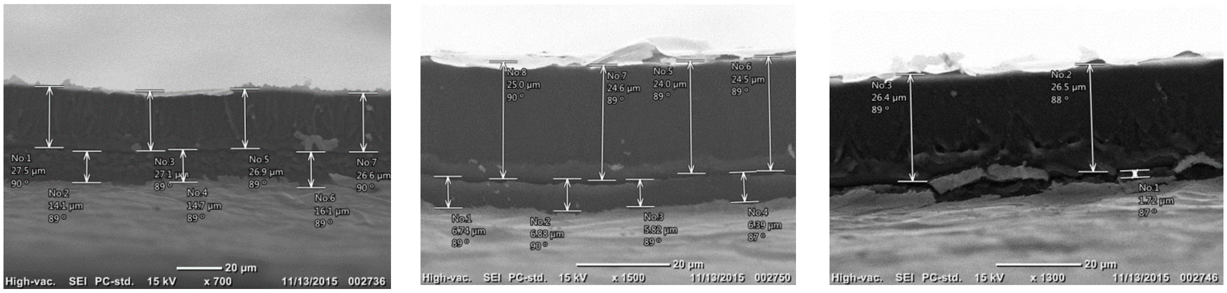

(a) (b) (c)

Figure S1: SEM images of specimen Nafion/1s-PEDOT:PSS/Au, from middle point to edge ((a) - (c)) of PEDOT:PSS layer.

For the bilayer laminate with much larger length respect to its width and thickness, the elastic modulus of each layer can be deducted as described by Liu *et al*. previously [1]. Figure S2 illustrates a bilayer laminate with the length much larger than the other dimensions. The elastic modulus of the entire structure Y^e is dependent on the elastic modulus of each layer Y^a and Y^b as:

$$Y^e = aY^a + bY^b \qquad (1)$$

where a and b are the volume fractions of the corresponding layer in the laminate structure.[1]

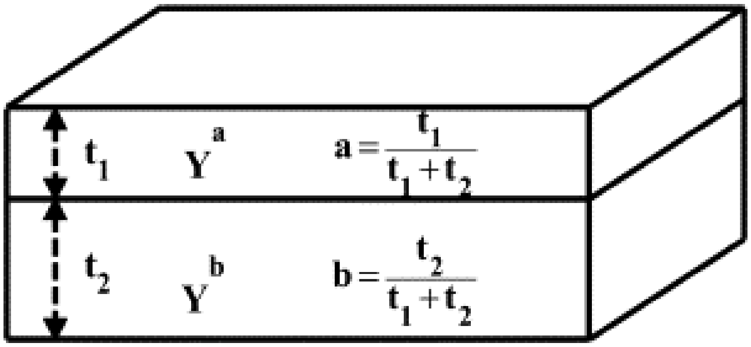

Figure S2: Schematic of a bilayer laminate for the characterization of the elastic modulus of individual layer. [1]

The thickness of the each layer is read from the SEM images; average value from the middle point to the edge of the PEDOT:PSS layer was taken for the average thickness. Three tensile tests were conducted to take the average value. The elastic modulus of each component measured, deduced or read from other literatures is listed in Table S1, with the corresponding Poisson's ratio listed in the next column.

Table S1: The thickness of each layer in IEAP actuator and its physical properties.

Sample	Layer	Thickness (μm)	Elastic Modulus (MPa)	Poisson's Ratio
Nafion/1s-PEDOT:PSS	overall	40.2	20.7	
	Nafion	28.6	27.3	0.487[2]
	PEDOT:PSS	11.6	4.3	0.33[3]
Nafion/1s-PEDOT:PSS/Au	gold leaf	0.05	20,000[1]	0.42[4]

2. Simulation of electromechanical response by FEM

FEM is performed to model the electromechanical response of IEAP actuators with different patterns. The mechanical deformation is modeled by ABAQUS finite element code. Due to the

pretty small width of the IEAP actuator (1 mm), the normal stress and the shear stresses directed perpendicular to the plane in which the bending occurs are assumed to be zero. As a result, the 3-D configuration of IEAP actuator can be reasonably approximated as a 2-D plane stress configuration in the preprocessing module, with a 4-node bilinear plane stress quadrilateral (CPS4R) element for the analysis. Moreover, SEM images reveal a non-uniform distribution of PEDOT:PSS layer, whose highest concentration is in the center, and gradually tapers off along the edge. A trapezoid-like geometry is used to represent the PEDOT:PSS pattern. Material's property and geometry of each main component are read from SEM images and Table S1. Tie constraint is employed to model the surface contact with gold electrode as master surface and Nafion with PEDOT:PSS pattern as slave surface, with the assumption that no relative displacement happened due to the hot-pressed bonding. The boundary condition of ENCASTRE (U1 = U2 = U3 = UR1 = UR2 = UR3 = 0) is adopted at one end of the model to represent the mechanically fixed end of the actuator.

The actuation response of IEAP actuators with ions from EMI-Tf in this study is caused by i) the accumulation and depletion of excess charges at the electrodes, and ii) the expansion of the coated PEDOT:PSS layer when under an applied voltage, which are both equivalent to a thermal bimorph in mechanism. Nafion membrane is divided into four layers along the thickness evenly. The layer connected to cathode and anode are referred to as Nafion/cat and Nafion/ani, respectively. These two layers are used to simulate the expansion and contraction due to ions accumulation and depletion at different electrodes. The other two layers located in the middle are named by Nafion/neu, to simulate the ions depletion during the actuation process. A consistent isotropic thermal coefficient α_L is applied to each main component, and temperature field is used to control the deformation of each layer. In ABAQUS the definition of the isotropic thermal coefficient α_L is

the ratio of change in length (ΔL) to the total starting length (L) and change in temperature (ΔT), with the expression as $\Delta L/L = \alpha_L \times \Delta T$. The change in area of the 2D plane cross-section due to thermal expansion is $\Delta A = hl \times (1 + \alpha_L \times \Delta T)^2 - hl \approx 2hl.\alpha_L \times \Delta T$ if we exclude the higher orders due to the pretty small value of ΔL. As a result, the change in area (ΔA) caused by thermal expansion/contraction is linear to the change in temperature (ΔT). Same approximation is also applicable to the change in volume (ΔV).

Define N as the total amount of cations drifted in the cationic response of actuation. Following paragraphs introduce the detailed procedures and their theoretical support.

1. Actuator 1S

a) Cationic response: N cations are drifted from Nafion/neu and Nafion/ani layers homogeneously, and accumulated in PEDOT:PSS layer (attached to cathode) and Nafion/cat layer with volume ratio of 2:1.
b) Anionic response: N anions/anionic clusters are drifted and stored in Nafion/ani layer from Nafion/cat and Nafion/neu layers homogeneously. The cations stored in PEDOT:PSS layer (attached to cathode) in previous cationic response won't move out during the anionic response.

The procedures adopted in actuator 1S are based on the observation of a non-ignorable expansion from PEDOT:PSS layer when it only exists on the convex side of Nafion. Meanwhile, IEAP actuator with bare Nafion also displays a noticeable bending, indicating ions accumulation at the outer layers of Nafion and depletion at the inner layers at the same time [1, 5]. In addition, PEDOT:PSS layer does not contain any ions at the very beginning, thus it won't show any contraction during the simulation.

2. Actuator 2SA

a) Cationic response: N cations are drifted from Nafion/neu and Nafion/ani layers homogeneously, and accumulated in PEDOT:PSS layer (attached to cathode) and Nafion/cat layer with volume ratio of 2:1.

b) Anionic response: N anions/anionic clusters are drifted from Nafion/neu and Nafion/cat homogeneously. Then, (i) part of the anions/anionic clusters are stored in PEDOT:PSS layer (attached to anode) with the same volume density of the cations in PEDOT:PSS layer (attached to cathode) in the previous cationic response, and (ii) remaining anions/anionic clusters are accumulated in Nafion/ani layer.

The procedures adopted in the simulation of actuator 2SA are based on the same reason with part 1.

3. Actuator 2SS

Actuator 2SS differs remarkably from actuator 1S and 2SA. There are five segments along the length, with 3 segments made of Nafion/2s-PEDOT:PSS and 2 segments made of BNafion. Due to their significant difference in electrochemical and electromechanical responses, a segment-wise procedure is employed as below:

a) Cationic response: in each segment made of BNafion, n (= N/5) cations are drifted from Nafion/neu and Nafion/ani layers homogeneously, and accumulated in Nafion/cat layer. In each segment made of Nafion/2s-PEDOT:PSS, n/8 cations are drifted from Nafion/neu and Nafion/ani layers homogeneously, then accumulated in PEDOT:PSS layer and Nafion/cat layer with volume ratio of 2:1.

b) Anionic response: in each segment of BNafion, n anions/anionic clusters are drifted and stored in Nafion/ani layer from Nafion/cat and Nafion/neu layers homogeneously. In each segment made of Nafion/2s-PEDOT:PSS, n=8 anions/anionic clusters are drifted from Nafion/neu and

Nafion/cat homogeneously. Then, (i) part of the anions/anionic clusters are drifted and stored in PEDOT:PSS layer(attached to anode) with the same volume density of the cations stored in PEDOT:PSS layer (attached to cathode) in the previous cationic response, and (ii) remaining anions/anionic clusters are accumulated in Nafion/ani layer.

The procedures adopted in actuator 2SS are based on the observation from the equivalent circuit modeling. Specimen Nafion/2s-PEDOT:PSS reveals a much smaller EDL capacitance (0.12 μF) at the electrode surface when compared to the specimen BNafion (2.86 μF). As a result, BNafion and Nafion/2s-PEDOT:PSS are treated differently for a best match of the experimental results.

From the equivalent circuit modeling, the ratio of EDL capacitance of specimen Nafion/2s-PEDOT:PSS and specimen BNafion is 0.12/3.24 = 0.037, while from the simulation procedures above, this ratio becomes 1/8 = 0.125, in order to fully match the experimental results. It suggests the complexity to quantify the ions/ion clusters motion during the actuation. However, all the procedures made above is consistent with our previous electrochemical and electromechanical responses, and fully respect to the conclusions in earlier works.[1, 5, 6]

References for supporting information

[1] S. Liu, R. Montazami, Y. Liu, V. Jain, M. Lin, X. Zhou, J. Heflin, Q. Zhang, Sensors and Actuators A: Physical 2010, 157, 267.

[2] J. Y. Li, S. Nemat-Nasser, Mechanics of materials 2000, 32, 303; H.-C. Wei, G.-D. J. Su, Sensors 2012, 12, 11100.

[3] F. Greco, A. Zucca, S. Taccola, A. Menciassi, T. Fujie, H. Haniuda, S. Takeoka, P. Dario, V. Mattoli, Soft Matter 2011, 7, 10642.

[4] T. E. T. Box.

[5] R. Montazami, S. Liu, Y. Liu, D. Wang, Q. Zhang, J. R. Heflin, 2011.

[6] Y. Liu, S. Liu, J. Lin, D. Wang, V. Jain, R. Montazami, J. R. Heflin, J. Li, L. Madsen, Q. Zhang, Applied Physics Letters 2010, 96, 223503; Y. Liu, R. Zhao, M. Ghaffari, J. Lin, S. Liu, H. Cebeci, R. G. de Villoria, R. Montazami, D. Wang, B. L. Wardle, Sensors and Actuators A: Physical 2012, 181, 70.
